# Low-temperature gas from marine shales

**DOI:** 10.1186/1467-4866-10-3

**Published:** 2009-02-23

**Authors:** Frank D Mango, Daniel M Jarvie

**Affiliations:** 1 Petroleum Habitats, 806 Soboda Ct., Houston, Texas, 77079, USA; 2 Worldwide Geochemistry, 218 Higgins Street, Humble, Texas, 77338, USA

## Abstract

Thermal cracking of kerogens and bitumens is widely accepted as the major source of natural gas (thermal gas). Decomposition is believed to occur at high temperatures, between 100 and 200°C in the subsurface and generally above 300°C in the laboratory. Although there are examples of gas deposits possibly generated at lower temperatures, and reports of gas generation over long periods of time at 100°C, robust gas generation below 100°C under ordinary laboratory conditions is unprecedented. Here we report gas generation under anoxic helium flow at temperatures 300° below thermal cracking temperatures. Gas is generated discontinuously, in distinct aperiodic episodes of near equal intensity. In one three-hour episode at 50°C, six percent of the hydrocarbons (kerogen & bitumen) in a Mississippian marine shale decomposed to gas (C_1_–C_5_). The same shale generated 72% less gas with helium flow containing 10 ppm O_2 _and the two gases were compositionally distinct. In sequential isothermal heating cycles (~1 hour), nearly five times more gas was generated at 50°C (57.4 μg C_1_–C_5_/g rock) than at 350°C by thermal cracking (12 μg C_1_–C_5_/g rock).

The position that natural gas forms only at high temperatures over geologic time is based largely on pyrolysis experiments under oxic conditions and temperatures where low-temperature gas generation could be suppressed. Our results indicate two paths to gas, a high-temperature thermal path, and a low-temperature catalytic path proceeding 300° below the thermal path. It redefines the time-temperature dimensions of gas habitats and opens the possibility of gas generation at subsurface temperatures previously thought impossible.

## Background

The hydrocarbons in natural gases are believed to come from two sources, one biological ('biogenic gas'), and the other from thermal cracking, 'primary thermal gas' from kerogen cracking, and 'secondary thermal gas' from oil cracking [1,2]. Thermal cracking is a high-energy endothermic reaction that generates gas between 100 and 200°C in the subsurface [2] and generally above 300°C in the laboratory [3-13]. There are examples of gas deposits possibly generated at lower temperatures [14-19], and reports of gas generation over long periods of time at 100°C [20], but we are aware of no reports of gas generation at temperatures substantially below 100°C.

We addressed the possible existence of a low-temperature path to gas catalyzed by low-valent transition metals (LVTM) [21-24]. Such a path could have escaped detection in the past because it was suppressed at high temperatures and the oxic conditions of pyrolysis [3-13]. Oxygen is a powerful poison of LVTM [25] and organometallic catalysts, like ordinary hydrocarbons, decompose at temperatures above 300°C.

## Results and discussion

Here we report anoxic experimental procedures in which some marine shales generate gas at extraordinarily low temperatures (50°C). The gas differs in almost all respects from that generated at higher temperatures (> 300°C) through thermal cracking and strongly suggests the existence of a second, low-energy catalytic path to natural gas.

Under anoxic helium flow, most shales released between 1 and 1,000 μg C_1_–C_5_/(g shale) at 100°C (unpublished), and some released substantial amounts of hydrocarbons at 50°C (Table 1).

**Table 1:** Gas generation from marine shales under helium flow at 50°C; gas yields, gas compositions, and Rock Eval data.

Shale	F	F(Ox)	F	F	F	F	NA	B
**Depth (m)**	1552	1552	1578	1578*	1578*	1582	1025	6300

**Yield**	827	230	57	13	10	230	102	0

**CH_4_**	31	10	13	0	0	11	0	0

**C_2_H_6_**	29	32	34	23	11	30	27	0

**C_3_H_8_**	21	30	27	32	29	29	34	0

***i*-C_4_H_10_**	3.1	3.5	3.6	5.0	8.9	3.2	3.0	0

***n*-C_4_H_10_**	9.2	13	12	19	23	14	18	0

***i*-C_5_H_12_**	3.1	4.3	4.1	8.3	12	3.9	4.4	0

***n*-C_5_H_12_**	3.4	7.4	6.4	14	17	9.2	14	0

**TOC**	5.8	5.8	4.4	3.9	3.9	4.4	10.2	9.4

**S1**	2.1	2.1	2.3	1.5	1.5	1.6	3.4	7.5

**S2**	10.8	10.8	10.5	8.8	8.8	8.72	32.5	61.1

**S3**	0.46	0.46	0.39	0.35	0.35	0.29	0.29	2.45

**Tmax**	449	449	450	449	449	448	445	434

**Figure 1:**
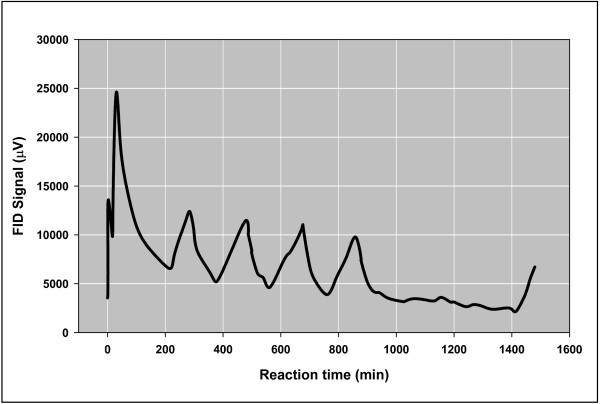
**Gas generation from Floyd shale under helium flow at 50°C for 24 hours**. The effluent gas was passed through an ice trap (1/4 inch copper tubing), then directly into a flame ionization detector (FID) where the signal was recorded over 24 hours. The sample was a Mississippian Floyd shale (well cuttings) from a well in the Black Warrior Basin in Clay County, Mississippi (API = 23025200660000; +33.79, -88.820; 1582 m); Rock-Eval, TOC = 4.4; S1 = 1.6; S2 = 8.7; S3 = 0.29; Tmax = 448. About 0.18 mg hydrocarbons/(g shale) was generated over 24 hours based on the integrated FID peaks calibrated with a standard mixture.

**Figure 2:**
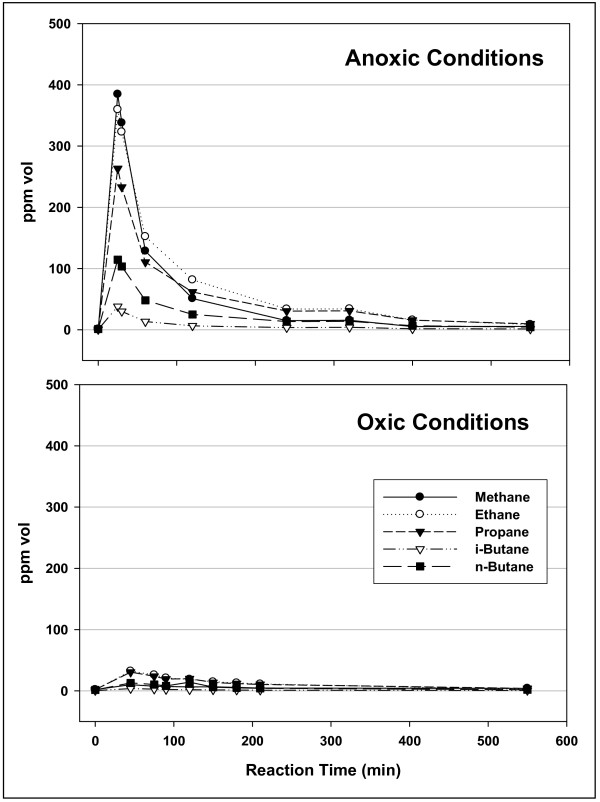
**The C_1_–C_4 _hydrocarbons produced from Floyd shale under helium flow at 50°C**. The procedure (Anoxic Conditions) in Fig. 1 was repeated with another sample of Floyd shale at 1552 m. Products were periodically withdrawn from the reactor effluent gas stream and analyzed by GC. Gas compositions are concentrations (ppm vol) in the effluent gas stream over time. Under Oxic Conditions, an aliquot of the same shale was ground to 60 mesh in air, the reactor was not pressure flushed with pure helium, and gas flow at 50°C employed helium with 10 ± 1 ppm O_2_. Rock-Eval (before anoxic reaction) TOC = 5.78; Tmax = 449; S1 = 2.09; S2 = 10.8; S3 = 0.46. Rock-Eval (after anoxic reaction) TOC = 3.93; Tmax = 451; S1 = 1.84; S2 = 10.37; S3 = 0.45. Yields (integration): 0.83 mg C_1_–C_5_/g (Anoxic); 0.23 mg C_1_–C_5_/g (Oxic). Ground samples were injected directly into a 300°C chamber under helium flow in Rock-Eval analysis. Thus, any C_1_–C_5 _hydrocarbons desorbed under helium flow at 50°C in our experiments would have been integrated into the Rock-Eval S1 peak.

**Figure 3:**
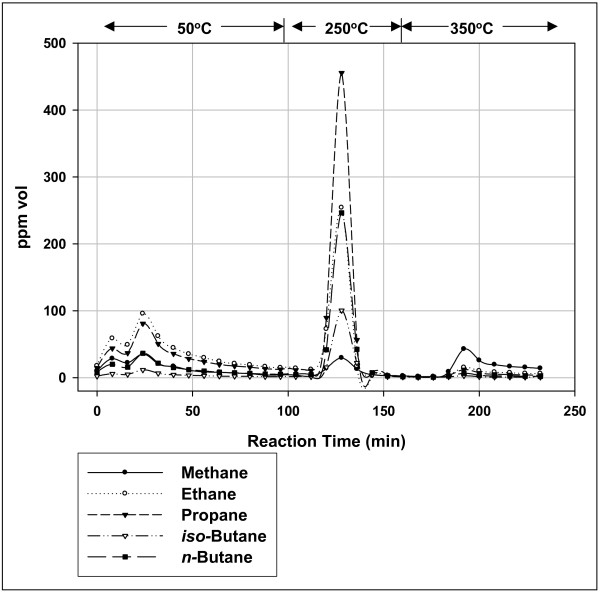
**Gas compositions from a marine shale under isothermal anoxic helium flow in sequential heating cycles at 50°C, 250°C, and 350°C**. Sample: the Floyd shale in Fig. 1 at 1578 m; TOC = 4.42; S1 = 2.3; S2 = 10.5; S3 = 0.39; Tmax = 450. Yield: 57.4 μg (C_1_–C_5_)/(g rock) at 50°C; 78.9 μg (C_1_–C_5_)/(g rock) at 250°C; 12 μg (C_1_–C_5_)/(g rock) at 350°C. The C_1_–C_5 _products contained no olefins below 350°C, and 33% vol C_2_–C_4 _olefins at 350°C. C_5_olefins were not isolated in our analytical procedure. Olefins are not included in Yield.

Yield in μg (C_1_–C_5_)/g rock was calculated by integration (calibrated by C_1_–C_5 _standard mixture). Gas compositions are % vol. TOC is total organic carbon as %; S1 = free hydrocarbons distilled from the rock (300°C) in mg/g rock; S2 = cracked hydrocarbons (350 – 550°C) in mg/g rock; S3 = carboxyl decomposition products (300 – 390°C) in mg CO_2_/g rock; Tmax is the temperature (°C) of the S2 peak. The Floyd shale (F) is described in Fig. 1. The New Albany shale (NA) (Dev/L Miss., Illinois Basin) is side wall core from a well in Union County, Kentucky (API = 16225974700000; +37.565, -88.076; 1025 m). The Bakken shale (B) is U Dev/L Miss whole core from a well in Stark County, North Dakota (API = 33089004240000; +46.887; -102.882; 6300 m) in the Williston Basin. F(Ox) represents Floyd shale under Oxic Conditions in Fig. 2. The reactions with New Albany, Bakken, and Floyd shale at 1582 m are duplicates of that in Fig. 2, and the reaction with Floyd shale at 1578 m is shown in Fig. 3 (80 min., 50°C). Samples were between 1 and 2 gm with particle sizes (before grinding) generally between 2 and 5 mm except for the duplicate Floyd experiments (1578*) (80 min., 50°C) which used aliquots of a mixture of particle sizes under 2 to 5 mm. The Bakken core had been in storage 15 years, the Floyd cuttings in storage over 3 years, and the New Albany shale in storage 3 years.

They were hydrocarbons *generated *under gas flow as opposed to desorbed pre-existing hydrocarbons:

1) Gas release curves (concentrations vs time) were not desorption curves. Isothermal desorption curves under gas flow are first order with concentrations falling exponentially over time [26]. Gas was released discontinuously in our experiments. It occurred in discrete irregular episodes of near uniform intensity continuing for long periods of time. Fig. 1 shows a typical example. A Mississippian marine shale (Floyd, Black Warrior Basin) released 180 μg gas/(g shale) in five distinct three-hour episodes over 24 hours under anoxic helium flow at 50°C. Nonlinear kinetic behavior like that in Fig. 1 often attends chaotic chemical reactions defined by Field and Györgyi [27] as "oscillatory but aperiodic, apparently random behavior appearing in a system not subject to stochastic perturbation but entirely governed by a deterministic dynamic law." Chaotic catalytic reactions are reported in transition metal catalytic oxidation of carbon monoxide, ammonia, hydrocarbons, and nitric oxide, and in the hydrogenation of olefins, carbon monoxide, and nitric oxide [28-30]. We are aware of no reports of episodic desorption or ejections from inclusions under isothermal gas flow. Such hypothetical processes should display broad overlapping peaks quite distinct from those in Fig. 1.

2) The Floyd shale in Fig. 2 desorbed only 0.25 mg free hydrocarbons over the course of reaction (S1 Rock-Eval peak before and after the run), but it released 0.83 mg C_1_–C_5_/g in the experiment (3 hours, 50°C). Since our Rock-Eval analysis would include any C_1_–C_5 _hydrocarbons in the S1 peak, desorption of pre-existing light hydrocarbons can only account for a small fraction of the gas released in this experiment.

3) Trace levels of oxygen (helium with 10 ppm O2) suppressed hydrocarbon release and altered gas compositions consistent with catalyst poisoning [25]. The upper panel of Fig. 2 shows the distribution of hydrocarbons generated in one three-hour episode under anoxic helium flow. An aliquot of the same shale generated 72% less gas under helium flow with 10 ppm oxygen (lower panel, Fig. 2), and the two gases were distinct. The gas generated under oxic conditions contained 10% vol methane while the anoxic procedure generated a gas with 31% vol methane (Table 1). Substantially more gas was generated at 50°C under anoxic conditions (75 mg C_1_–C_5_/g kerogen) than is typically generated by type II kerogen cracking at 350°C for comparable time periods: average ~5 mg C_1_–C_5_/g kerogen, non-isothermal 200 to 350°C [13]; 20 mg C_1_–C_5_/g kerogen, isothermal 350°C [10].

Not all shales analyzed in our experiments generated gas at 50°C and we saw large variations between different shales in the amounts of gas generated and in their compositions. Table 1 shows the differences between Floyd shales at different depths (same well), New Albany shale from the Illinois Basin and Bakken shale from the Williston Basin. Sample size (before grinding) had an effect on gas generation. In the more productive shales (Floyd and New Albany), particles 2 – 5 mm and larger would generate gas, while smaller samples, possibly oxidized, generally would not. We attempted to use samples of uniform size (2 – 5 mm) in our experiments. Aliquots of homogeneous mixtures were used in the comparative experiments in Fig. 2 and in the duplicate experiments in Table 1 (Floyd 1578*). We attribute part of the differences between the Floyd experiment at 1578 m generating 57 μg gas/g and the subsequent duplicate experiments (Floyd 1578* m) generating 10 and 13 μg gas/g to differences in particle size. The first experiment used particles in the 2–5 mm range while the duplicate experiments used the smaller pieces remaining. The duplicate experiments are included in Table 1 to illustrate the analytical sensitivity to sample selection (particle size) and analytical reproducibility with samples of uniform composition (aliquots).

Figure 3 illustrates the differences between gas generation at low and high temperatures in a single experiment. In three sequential heating cycles (50°C, 250°C, and 350°C), substantially more gas was generated at low temperatures than at thermal cracking temperature (350°C) and the gases differed sharply (Table 2). The gas at 350°C contained ~30% vol olefins while the low-temperature gases contained no olefins. The high-temperature gas was similar in composition to thermogenic gas generated from type II kerogen pyrolysis under similar conditions [10], suggesting that it is largely thermogenic. The low-temperature gases are probably not thermogenic. It is more likely that they were generated along a different pathway controlled by nonlinear kinetics, a path that does not generate olefins. We have seen no evidence of episodic gas generation at 350°C suggesting that the second pathway no longer functions at higher thermal cracking temperatures.

**Table 2:** Gas compositions (% vol) of C_1_–C_5 _saturates in Figure 3.

Temperature	Methane	Ethane	Propane	*Iso*-Butane	*n*-Butane	*iso*-Pentane	*n*-Pentane
50°C	13	34	27	3.6	12	4.1	6.4

250°C	3.5	18	32	7.7	18	9.3	11

350°C	48	19	14	3.2	7.6	3.7	5.4

The differences in yield and gas composition in Fig. 3 (Table 2) are significant because they are from one sample in a single reaction. The differences are therefore intrinsic to the shale at different temperatures independent of sample composition or analytical procedure (e.g., sample preparation or flow conditions). The Floyd sample generated two sharply different gases, the major gas at low temperatures below 300°C, and the minor gas at thermal cracking temperatures above 300°C.

There is no clear relationship between the amounts of low-temperature gas generated and the organic carbon content of the shale (S1 and S2 in Rock-Eval). Different shales showed large variations in gas yield and gas composition, and there were large differences between Floyd shales at different depths from the same well, but the differences were largely independent of organic carbon content (Table 1).

Floyd shale generated about 4 times more gas under anoxic conditions than under oxic conditions (Fig. 2), and about 10 times more gas below 300°C than above (Fig. 3). This is consistent with a catalytic reaction with a low activation energy (low-energy path) proceeding at low temperatures, and a thermal reaction with a high activation energy (high-energy path) proceeding perhaps exclusively at high temperatures.

Other explanations are less plausible. The arguments against desorption have already been discussed. It cannot explain nonlinear kinetics, the amounts of hydrocarbons released, or the effects of oxygen. Trace levels of oxygen could oxidize trace levels of catalyst [25], thereby suppressing the generation of much larger amounts of catalytic gas. It cannot be acting stoichiometrically, through hydrocarbon oxidation, for example. 1.07 μmole O_2 _(200 minutes of He flow with 10 ppm O_2_) had to account for 0.6 mg gas/(g shale) missing under oxic conditions. But this much oxygen could only oxidize 10 μg hydrocarbon to CO_2 _+ H_2_O, less than 2% of the missing gas.

The possibility that our reactors could be catalytic is also unlikely. No detectable amounts of gas were generated in blank experiments with clean sands impregnated with *n*-octadecane. Only the addition of marine shales under anoxic conditions resulted in robust gas generation and not all shales generated hydrocarbons. A Bakken shale with high concentrations of free hydrocarbons (S1 = 7.5 mg/g) generated < 1 μg C_1_–C_5_/g while Floyd shale with substantially less free hydrocarbons (S1 = 2.1 mg/g) generated nearly one thousand times more gas under the same conditions (Table 1). This is consistent with Floyd shale generating C_1_–C_5 _hydrocarbons and inconsistent with the Floyd shale releasing adsorbed hydrocarbons which were subsequently converted to C_1_–C_5 _on the reactor's surface. The reaction generated 830 μg C_1_–C_5_/g but the Floyd shale desorbed only 250 μg hydrocarbons/g over the course of reaction.

Thermal cracking and biogenesis are also unlikely. First-order thermal cracking would not generate the nonlinear curves in Figs. 1, 2, 3 and robust thermal cracking at 50°C is unprecedented. There is the possibility of biological activity generating gas at 50°C, and even as high as 130°C [31], but ethanogens and propanogens are rare and biogenic gas at 250°C is unlikely (Fig. 3).

Free energy barriers at low temperatures make it difficult to explain gas generation through thermal cracking, even with catalytic assistance. A catalyst can bring a reaction to equilibrium, but it cannot alter the free energy change. Butane, for example, will crack to insignificant amounts of methane and propene (eq. 1) at temperatures below 300°C because ΔG is positive at these temperatures [32].

(1) C_4_H_10 _→ CH_4 _+ C_3_H_6_

Our results suggest a different reaction path. Decomposition to carbon and gas is one possibility that is energetically very favorable at low temperatures. For example, ΔG for butane decomposition (eq. 2) (C denotes carbon in unspecified form) is – 15.9 kcal/mol at 25°C compared to +6.94 kcal/mol for eq. 1 at 25°C.

(2) C_4_H_10 _→ CH_4 _+ C_2_H_6 _+ C

We see no olefins at 50 and 250°C consistent with catalytic decomposition through eq. 2 and substantial amounts of olefins at 350°C consistent with thermal decomposition through eq. 1 proceeding almost exclusively at high temperatures.

The marine shales analyzed here would appear to be naturally catalytic and LVTM are outstanding possibilities. Their partially filled *d *orbitals would account for the high activity [33,34], sensitivity to oxygen poisoning [25], and nonlinear kinetics [27-30]. They have been suggested as possible catalysts in natural gas generation [21], and this hypothesis has received laboratory support [22,35-38].

Natural activity may have escaped attention until now because of the conditions employed in simulation experiments and because unexpected results can be overlooked. Kerogens are often isolated from their inorganic host rocks by chemical digestion in air [39] and procedures rarely excluded oxygen in sample preparation and analysis [3-13]. Most procedures were at temperatures above 300°C where catalyst decomposition could occur.

Gas generation at ambient temperatures may have occurred in the past, but was not recognized as such [40]. In experiments to determine the amounts of gas lost from cuttings while in storage (months), workers encountered one shale (White Specs) that "actually yielded more gas as the length of the storage period increased". Gas was analyzed by grinding the shales to fine powders in closed containers. It was thus internal gas that was analyzed as opposed to external adsorbed gas which was probably lost in storage. Although biogenic gas from contamination is always a possibility, the samples were fresh shales in which internal contamination would seem improbable. It is more likely that the interior anoxic surfaces of White Specs shale were uncontaminated with surface biota and generated gas while in storage in much the same way that the inner anoxic surfaces of Floyd and New Albany shales generated gas in our experiments.

## Experimental

Our objective was to analyze the inner anoxic surfaces of marine shales for evidence of natural catalytic activity by LVTM. Knowing their high sensitivity to oxygen poisoning, we adopted an analytical procedure in which oxygen was rigorously excluded (anoxic conditions). Helium was purchased as 'high purity' and further purified through commercial oxygen scrubbers. Helium with 10 ± 1 ppm vol oxygen was used in experiments designated 'oxic'. Shale samples were ground to 60 mesh with mortar and pestle in plastic glove bags filled with high-purity argon. Freshly ground powders (0.5 to 3 g) were transferred from glove bags to 5 ml (diameter = 1.27 cm) tubular brass reactors secured at each end to 1/4 inch copper tubing through Swagelok fittings. New reactors were constructed for most experiments. Weighed samples were transferred into reactors in air taking care to minimize time of exposure. The tubing was attached to gas lines through valves to open and close the system to gas flow. Reactors were flushed with flowing gas (helium, 12 ml/min) for 10 minutes at room temperature to remove the air picked up in reactor assembly. To remove any light hydrocarbons released in grinding and the remaining oxygen, they were then pressure flushed (purified helium) five times at ambient temperatures by pressurizing to 0.3 MP and venting to the atmosphere. Reactors (now anoxic) were heated (12.5°C/min) under purified helium flow (~0.3 MP; 12 ml/min) to reaction temperatures, where gas flow was continued at constant temperatures and the products (methane through pentane, C_1_–C_5_) analyzed over time by standard gas chromatography using a 50 m × 0.20 mm, 0.50 μm HP-PONA 19091S-001 column purchased from Agilent. The effluent gas stream was passed through a 1/4 inch ice trap and then directly into a flame ionization detector where the hydrocarbons released from the shale could be monitored over the course of reaction.

## Conclusion

Marine shales possess natural catalytic activity for converting hydrocarbons (kerogens and bitumens) to gas at low temperatures. It raises the possibility of gas generation in low-maturity sedimentary rocks, places often ignored in the search for natural gas.

## Competing interests

The authors declare that they have no competing interests.

## Authors' contributions

Both authors made significant contributions to this work.
